# Unmasking Differential Effects of Rosiglitazone and Pioglitazone in the Combination Treatment with *n*-3 Fatty Acids in Mice Fed a High-Fat Diet

**DOI:** 10.1371/journal.pone.0027126

**Published:** 2011-11-03

**Authors:** Vladimir Kus, Pavel Flachs, Ondrej Kuda, Kristina Bardova, Petra Janovska, Michaela Svobodova, Zuzana Macek Jilkova, Martin Rossmeisl, Rui Wang-Sattler, Zhonghao Yu, Thomas Illig, Jan Kopecky

**Affiliations:** 1 Department of Adipose Tissue Biology, Institute of Physiology Academy of Sciences of the Czech Republic v.v.i., Prague, Czech Republic; 2 Research Unit of Molecular Epidemiology, Helmholtz Zentrum München, German Research Center for Environmental Health, Neuherberg, Germany; University of Pittsburgh, United States of America

## Abstract

Combining pharmacological treatments and life style interventions is necessary for effective therapy of major diseases associated with obesity, which are clustered in the metabolic syndrome. Acting via multiple mechanisms, combination treatments may reduce dose requirements and, therefore, lower the risk of adverse side effects, which are usually associated with long-term pharmacological interventions. Our previous study in mice fed high-fat diet indicated additivity in preservation of insulin sensitivity and in amelioration of major metabolic syndrome phenotypes by the combination treatment using *n*-3 long-chain polyunsaturated fatty acids (*n*-3 LC-PUFA) and rosiglitazone, i.e. an anti-diabetic drug of the thiazolidinedione (TZD) family. We investigated here whether pioglitazone, a TZD-drug in clinical use, could elicit the additive beneficial effects when combined with *n*-3 LC-PUFA. Adult male mice (C57BL/6N) were fed an obesogenic corn oil-based high-fat diet (cHF) for 8 weeks, or randomly assigned to various dietary treatments (i) cHF+F, cHF with *n*-3 LC-PUFA concentrate replacing 15% of dietary lipids; (ii) cHF+ROSI, cHF with 10 mg rosiglitazone/kg diet; (iii) cHF+F+ROSI; (iv) cHF+PIO, cHF with 50 mg pioglitazone/kg diet; and (v) cHF+F+PIO, or chow-fed. Plasma concentrations of 163 metabolites were evaluated using a targeted metabolomics approach. Both TZDs preserved glucose homeostasis and normal plasma lipid levels while inducing adiponectin, with pioglitazone showing better effectiveness. The beneficial effects of TZDs were further augmented by the combination treatments. cHF+F+ROSI but not cHF+F+PIO counteracted development of obesity, in correlation with inducibility of fatty acid β-oxidation, as revealed by the metabolomic analysis. By contrast, only cHF+F+PIO eliminated hepatic steatosis and this treatment also reversed insulin resistance in dietary obese mice. Our results reveal differential effects of rosiglitazone and pioglitazone, unmasked in the combination treatment with *n*-3 LC-PUFA, and support the notion that *n*-3 LC-PUFA could be used as add-on treatment to TZDs in order to improve diabetic patient's therapy.

## Introduction

Obesity-associated diseases, namely type 2 diabetes (**T2D**), dyslipidaemia and other morbidities clustered in the ‘metabolic syndrome’, predispose to cardiovascular disease and represent major health problem around the world [1], [2]. Complex ethiology of these diseases involves both genetic and environmental factors with a different contribution to disease progression in various individuals. To improve the efficacy of the therapy of above mentioned metabolic diseases, treatment strategies are typically based on the use of combined treatments with multiple mechanisms of action [3]–[5]. Such treatments optimally result in synergistic beneficial effects, while reducing dose requirements and the incidence of adverse side effects that are associated with most of the chronic pharmacological interventions. Concerning T2D, the major obesity-associated metabolic disease [6], thiazolidinediones (**TZDs**), namely rosiglitazone and pioglitazone, proved to be useful for the pharmacological treatment of hyperglycaemia in the patients, while used as add-on treatment to metformin [5] and other pharmaceuticals [7]. However, due to a relatively high risk of adverse cardiac effects, the use of rosiglitazone was banned by the Europe in 2010 [8], whereas it is increasingly restricted by the US Food and Drug Administration. Both TZDs may increase the risk of osteoporosis (reviewed in [8]) and enhance accumulation of body fat [9]. Pioglitazone but not rosiglitazone treatment improves blood lipid profile in diabetic subjects [10] while differently affecting HDL-cholesterol levels, as well as lipoprotein subclass particle concentrations and particle sizes [11]. However, even the safety of chronic pioglitazone therapy has been questioned by recent unfavourable evidence [8], [12]. Hence, a combination treatment, which would allow for a reduction in the effective dose of pioglitazone (or other prospective new drugs from the TZD family; see [13]), will be helpful for increasing the safety of the therapy of diabetic patients.

Since TZDs are specific agonists of peroxisome-proliferator activated receptor **(PPAR)** γ, the major transcriptional regulator in adipocytes, TZDs probably improve insulin sensitivity by (i) increasing lipid uptake and mitochondrial β-oxidation in adipose tissue [14], while reducing toxic effects of lipid accumulation in other tissues (lipotoxicity); (ii) affecting distribution of body fat through a decrease in the accumulation of abdominal fat and promoting hyperplasia of adipocytes in subcutaneous fat [9]; and (iii) inducing secretion of adiponectin [15], [16], i.e. the major adipokine with beneficial effects on insulin sensitivity. In addition, PPAR-γ-independent effects of TZDs have been described to occur in both muscle and liver, documenting a very complex mechanism of action of TZDs [15], [17], [18]. Moreover, also in agreement with the differential effects of pioglitazone and rosiglitazone on plasma lipids and lipoprotein observed in humans (see above), animal studies suggest some differences between pioglitazone and rosiglitazone in their effects on lipid metabolism. Thus, in white adipose tissue of *db/db* mice, rosiglitazone induced mitochondrial β-oxidation more strongly than pioglitazone [19], while *de novo* lipogenesis in mouse liver was preferentially stimulated by rosiglitazone [20]–[22]. In skeletal muscle, somehow conflicting results concerning modulation of β-oxidation by pioglitazone and rosiglitazone were found [23], [24].

Besides pharmacological interventions, lifestyle changes are inevitable in the treatment strategy of T2D patients and they are extremely important for prevention of the disease [25]. The patients may be advised [26] to increase their intake of *n*-3 long-chain polyunsaturated fatty acids **(LC-PUFA**), namely eicosapentaenoic acid (**EPA**; 20:5*n*-3) and docosahexaenoic acid (**DHA**; 22:6*n*-3), either by consuming more sea fish or by means of nutritional supplements. In humans, *n*-3 LC-PUFA act as hypolipidaemics and increase HDL levels, while reducing progression of atherosclerosis and cardiac events (reviewed in [27], [28]). The cardio protective effects were especially pronounced in patients with T2D who have had myocardial infarction [29]. Several studies in humans demonstrated that *n*-3 LC-PUFA supplementation could help to reduce obesity (reviewed in [30]). However, *n*-3 LC-PUFA could not revert insulin resistance in T2D patients [26], [31], [32]. Accordingly, in animal models, *n*-3 LC-PUFA intake prevented development of insulin resistance [21], [33]–[38], obesity, and dyslipidaemia [21], [37]–[41].

Both *n*-3 LC-PUFA [21], [38], [42], [43] and TZDs [44] reduce low-grade adipose tissue inflammation associated with obesity, as well as systemic inflammation [45] in both humans and experimental animals. Metabolic effects of *n*-3 LC-PUFA are largely mediated PPAR-α and PPAR-δ(-β), however liver X receptor-α, hepatic nuclear factor-4, and sterol regulatory element binding protein-1 are also involved [46], [47]. Besides acting directly as regulatory ligands, *n*-3 LC-PUFA also act through their active metabolites, eicosanoids [47], and other lipid mediators [48], and by inducing adiponectin [35]–[37]. The hypolipidaemic and anti-obesity effects of *n*-3 LC-PUFA probably depend on the suppression of lipogenesis and increase of fatty acid oxidation in the liver [37], as well as on the enhancement of mitochondrial biogenesis and β-oxidation in white fat [41] and intestine [49], but not in skeletal muscle [21], [38].

Our recent results in dietary obese mice demonstrated that rosiglitazone and *n*-3 LC-PUFA, both compounds administered at a relatively low dose, exerted additive effects in prevention and reversal of dyslipidaemia, low-grade inflammation of adipose tissue, and insulin resistance, reflecting the induction of adiponectin and synergistic improvement in muscle insulin sensitivity [21]. These results suggested that inclusion of *n*-3 LC-PUFA in TZD treatment may reduce dose requirements and incidence of the adverse side effects associated with TZDs. Prompted by the partially distinct biological effects of rosiglitazone and pioglitazone, we compared the effects of rosiglitazone and pioglitazone administered either singly or in combination treatments with *n*-3 LC-PUFA in mice fed a high-fat diet. Both TZDs prevented to some extent dyslipidaemia and impairment of glucose homeostasis and induced adiponectin. Rosiglitazone but not pioglitazone potentiated the anti-obesity effect of *n*-3 LC-PUFA, reflecting inducibility of fatty acid β-oxidation. The combination of TZDs with *n*-3 LC-PUFA improved the effects on plasma lipid profile and glucose homeostasis, which were stronger in case of pioglitazone and correlated with its marked anti-steatotic effects in the liver and with the induction of adiponectin.

## Results

### Prevention of obesity and anti-steatotic effects

**Figure 1:**
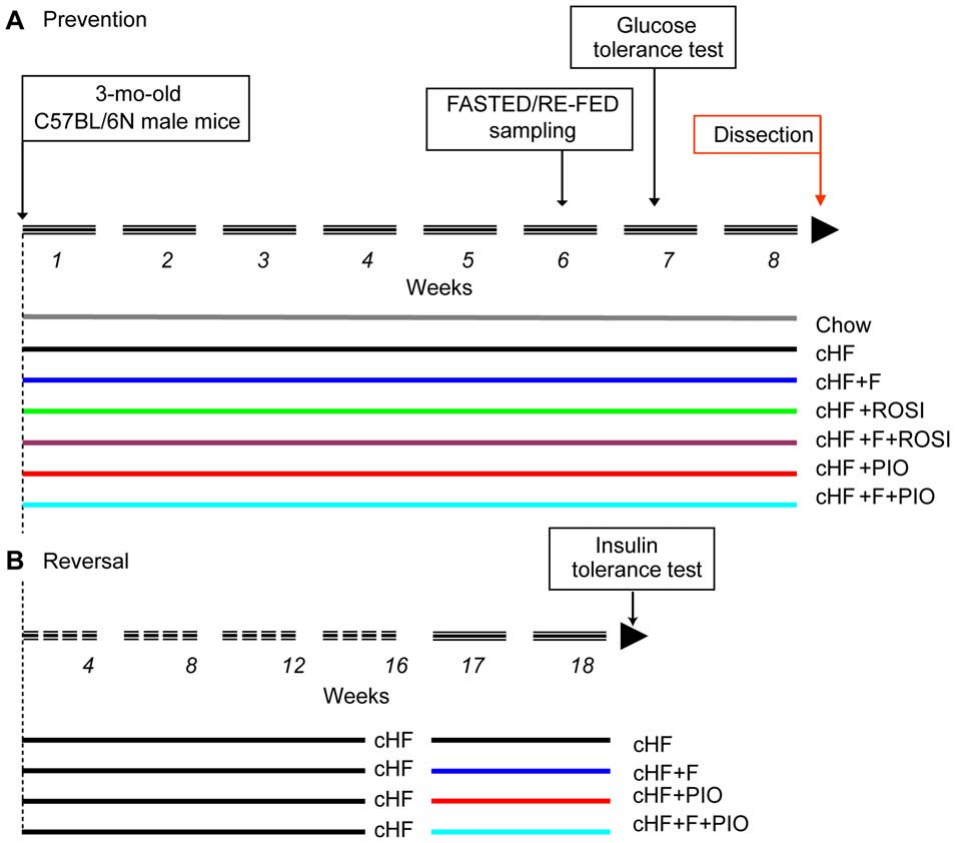
Overview of experimental setup.

**Figure 2:**
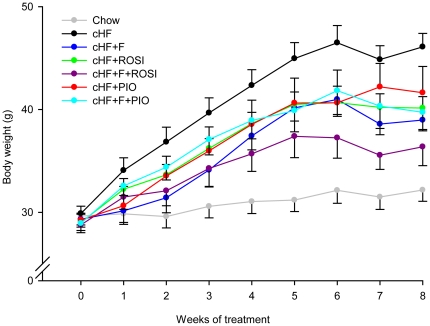
Body weights during differential dietary treatment. At 3 month of age, subgroups of mice were placed on cHF diet, or treated various cHF-based diets: cHF+F, cHF+ROSI, cHF+F+ROSI, cHF+PIO and cHF+F+PIO, or maintained on Chow diet for 8 weeks. Irregularities of the body weight caused by glucose tolerance test performed during week 7 (see Fig. 1) are apparent. Data are means ± SE (*n* = 7–8).

Three-month-old mice (see Fig. 1) were randomly assigned to a high-fat diet (lipid content ∼35% wt/wt, mainly corn oil; **cHF**) or to the following ‘treatments’ by (i) cHF diet supplemented with *n*-3 LC-PUFA concentrate replacing 15% wt/wt of dietary lipids (**cHF+F)**; (ii) cHF diet supplemented with 10 mg rosiglitazone/kg diet (**cHF+ROSI**); (iii) cHF diet supplemented with both *n*-3 LC-PUFA concentrate and rosiglitazone (**cHF+F+ROSI)**; (iv) cHF diet supplemented with 50 mg pioglitazone/kg diet **(cHF+PIO**); and (v) cHF diet supplemented with both *n*-3 LC-PUFA concentrate and pioglitazone **(cHF+F+PIO)**. While all the above groups were fed isocaloric diets containing the same total content of dietary fat, some mice were also fed standard laboratory diet (**Chow).** Mice fed cHF diet gained body weight much faster than those fed Chow (Fig. 2). Treatments by cHF+F, cHF+ROSI or cHF+PIO tended to counteract the obesogenic effect of cHF diet. As observed before [21], cHF+F+ROSI treatment reduced body weight gain even further. In contrast, no effect of pioglitazone used as add-on treatment to *n*-3 LC-PUFA (cHF+F+PIO) on body weight was observed (Table 1, Fig. 2). None of the treatments affected food consumption (Table 1).

**Table 1:** Growth characteristics, adiposity and lipid accumulation.

	Chow	cHF	cHF+F	cHF+ROSI	cHF+F+ROSI	cHF+PIO	cHF+F+PIO
Body weight (g)							
Initial	29.6±0.9	29.9±0.7	29.3 0.8	29.0±0.9	28.8±0.8	29.3±0.6	28.9±0.7
Final	32.1±1.3	46.5±1.7^f^	41.0±1.6	40.7±1.6	37.3±1.9^a^	40.7±3.2	41.8±2.3
Body weight gain	2.1±0.6	16.0±1.2^f^	11.7±0.8	11.5±1.2	8.1±1.3^a^	11.3±2.9	12.1±1.7
FC (kJ/day/animal)	72±4	72±2	69±2	70±2	65±2	71±2	65±1
Feed efficiency (mg BW/kJ)	3.2±0.6	27.4±2.7^f^	17.8±2.6^a^	20.6±1.6	16.7±1.3^a^	20.4±1.8	23.7±1.7
Fat depots weights (mg)							
Epididymal fat	783±129	3205±243^f^	2336±86	2270±263	2238±287	2289±479	2196±187
Mesenteric fat	348±22	1237±168^f^	711±78^a^	840±82	656±105^a^	868±146	607±10^a^
Subcutaneous fat	240±37	968±61^f^	732±84^a^	586±4^a^	404±111a,b	636±106^a^	636±83^a^
Leptin (ng/ml)	8±2	72±5^f^	53±4	58±10	46±6^a^	72±10	61±8
Triacylglycerol content (mg/g)							
Liver	31±3	87±10^f^	51±5^c^	145±29a,b,d	75±18^c^	100±18	34±4a,c,d,e
Muscle	18±3	37±7^f^	30±7	34±2	35±6	29±6	39±4
Hepatic *Scd-1*	1.10±0.06	0.19±0.07^f^	0.09±0.04	0.54±0.05a,b	0.34±0.11	0.40±0.11^b^	0.16±0.05^c^

Three-month-old mice were placed on various diets and killed after 8 weeks of the dietary treatment as described in Methods. Body weight and food consumption (FC; recorded weekly) data are related to the initial 6 weeks of the treatment; due to irregularities of the body weight and FC data caused by glucose tolerance test performed during week 7, data from the last 2 weeks of the treatment could not be included (see Fig. 1). Fat depot weights and levels of plasma leptin were recorded after the killing. Expression of SCD-1 gene in the liver was evaluated using quantitative RT-PCR analysis and standardized using elongation factor 1α (Gene ID: 13627). Data are means±SE (*n* = 7-8).

a Significantly different from cHF.

b significantly different from cHF+F.

c significantly different from cHF+ROSI.

d significantly different from cHF+F+ROSI.

e significantly different from cHF+PIO (ANOVA).

f Significantly different from Chow (t-test).

The changes in body weight could be explained by modulation of adiposity, as documented by weights of epididymal, mesenteric and subcutaneous fat depots, with only the subcutaneous fat reflecting the additive weight-reducing effects of the cHF+F+ROSI treatment (Table 1). Accordingly, only cHF+F+ROSI but not cHF+F+PIO treatment decreased plasma levels of leptin, the major adipokine, the levels of which reflect the magnitude of fat accumulation (Table 1). In both liver and skeletal muscle, cHF diet increased triacylglycerol content (Table 1). None of the treatments affected muscle lipid content. While cHF+F or cHF+F+ROSI treatments had no significant effects on hepatic lipid content, hepatic lipid content was increased by cHF+ROSI treatment as compared with the cHF mice. In contrast, hepatic lipid content was not affected by cHF+PIO treatment, and cHF+F+PIO treatment even lowered hepatic lipids to the Chow mice levels (Table 1). Both TZDs increased hepatic expression of the gene for stearoyl-CoA desaturase-1 (**SCD-1**) that is essential for lipogenesis (Table 1).

### Plasma lipid levels, glycaemia and metabolic flexibility

**Figure 3:**
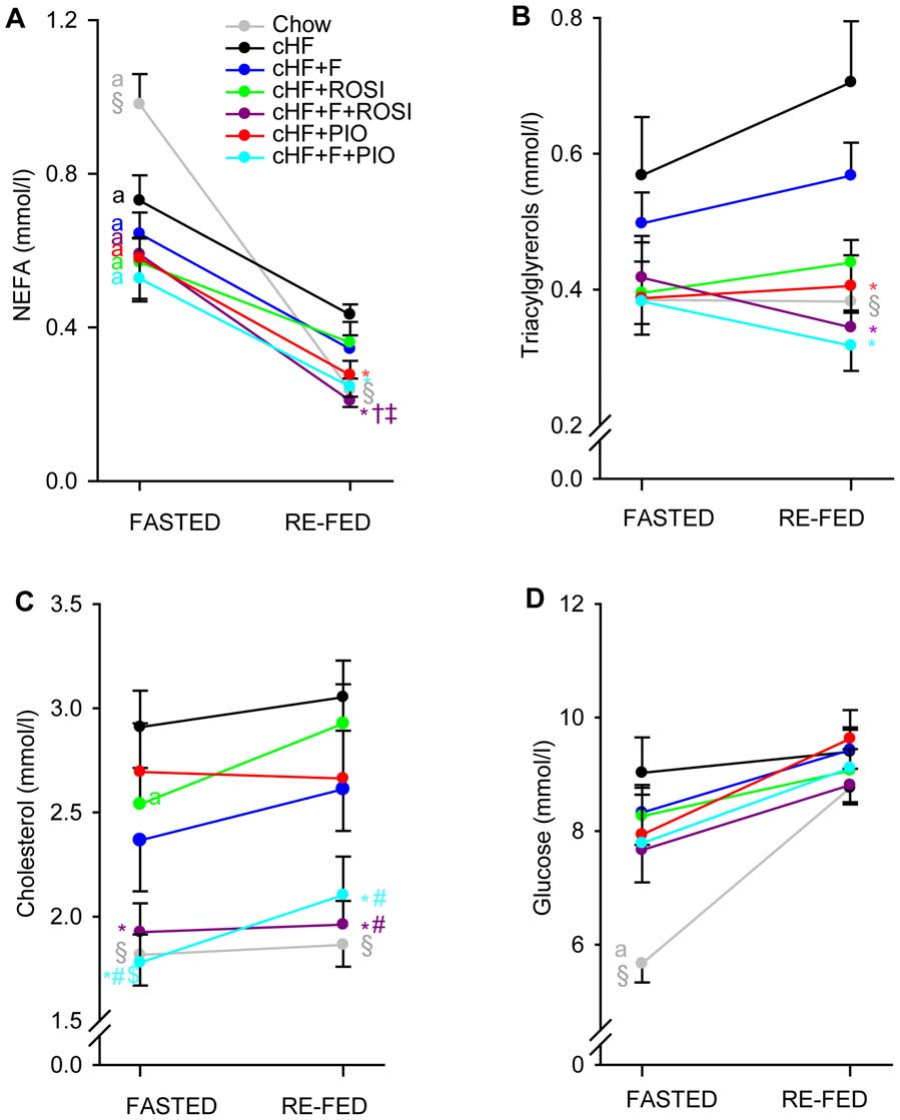
Plasmatic lipids levels and glycaemia during FASTED/RE-FED transition. At 3 month of age, subgroups of mice were placed on cHF diet, or treated various cHF-based diets. During week 6 of the treatment, plasma NEFA (**A**), triacylglycerols (**B**) and total cholesterol (**C**) levels and blood glucose (**D**) were assessed in FASTED and in RE-FED state as described in Methods in mice fed by Chow or cHF diet, or treated by cHF+F, cHF+ROSI, cHF+F+ROSI, cHF+PIO, and cHF+F+PIO diet. Data are means±SE (*n* = 7–8). ^a^ significantly different between FASTED and RE-FED state; *significantly different from cHF; ^†^significantly different from cHF+F; ‡significantly different from cHF+ROSI; ^#^significantly different from cHF+F+ROSI (ANOVA). ^§^Significantly different from cHF (t-test).

During week 6 (see Fig. 1), plasma levels of NEFA, triacylglycerols, and total cholesterol, and glycaemia were assessed in both FASTED and RE-FED state (i.e., in mice with the opposite but uniform feeding states, 3–4 hours after beginning of the dark phase of the day, when the feeding activity of all mice in the cohort should be normally high; see Methods; Fig. 3). In both states, higher levels of all these metabolic markers were detected in the cHF mice as compared with the Chow mice, reflecting the obesity and glucose intolerance developed in response to the high-fat feeding (see below), except for (i) FASTED NEFA levels, which were the highest in the Chow mice (Fig. 3A); and (ii) RE-FED glycaemia, with all the groups showing similar levels (Fig. 3D). Otherwise, all the treatments lowered the levels, or at least tended to decrease them, hence preserving to a various degree the ‘healthy’ metabolic profile (see also metabolomic analysis below) represented by the Chow mice. In most cases, the combination treatments (cHF+F+ROSI and cHF+F+PIO) resulted in the strongest effects, namely concerning the RE-FED triacylglycerol levels (Fig. 3) and the cholesterol levels under both metabolic states (Fig. 3C), and also the FASTED glycaemia tended to be suppressed the most in response to the combination treatments (Fig. 3D).

Metabolic adaptations to the FASTED/RE-FED transition are known to be deteriorated in obese and insulin-resistant states [1], [50]. Indeed, the cHF mice showed smaller changes in the levels of NEFA and glucose (Fig. 3A,D) and even opposite changes in the levels of triacylglycerols (Fig. 3B) in response to the FASTED/RE-FED transition as compared with the Chow mice. However, cholesterol levels remained stable in mice fed both Chow and cHF diet during the transition (Fig. 3C). None of the treatments preserved the NEFA response, with the exception of the cHF+F+ROSI treatment, which tended to normalize it (Fig. 3A). In contrast to NEFA, the triacylglycerol response was fully preserved by both combination treatments (cHF+F+ROSI and cHF+F+PIO; Fig. 3B). Single-type treatments (cHF+F, cHF+ROSI, and cHF+PIO) had no significant effect on the triacylglycerol response. The glycaemic response tended to be preserved by all the treatments, with only marginal differences between them (Fig. 3D).

Thus, the plasma lipid levels and glycaemia measured under both FASTED and RE-FED state, and the response of triacylglycerol levels to the FASTED/RE-FED transition, documented much better preservation of the ‘healthy’ metabolic profile by the combination treatments as compared with the single-type treatments. Only subtle differences between the effects of rosiglitazone and pioglitazone were observed, most consistently in case of the triacylglycerol-lowering effect, with pioglitazone showing a stronger effects in both single-type and cHF+F+PIO treatments (Fig. 3B).

### Glucose homeostasis

**Figure 4:**
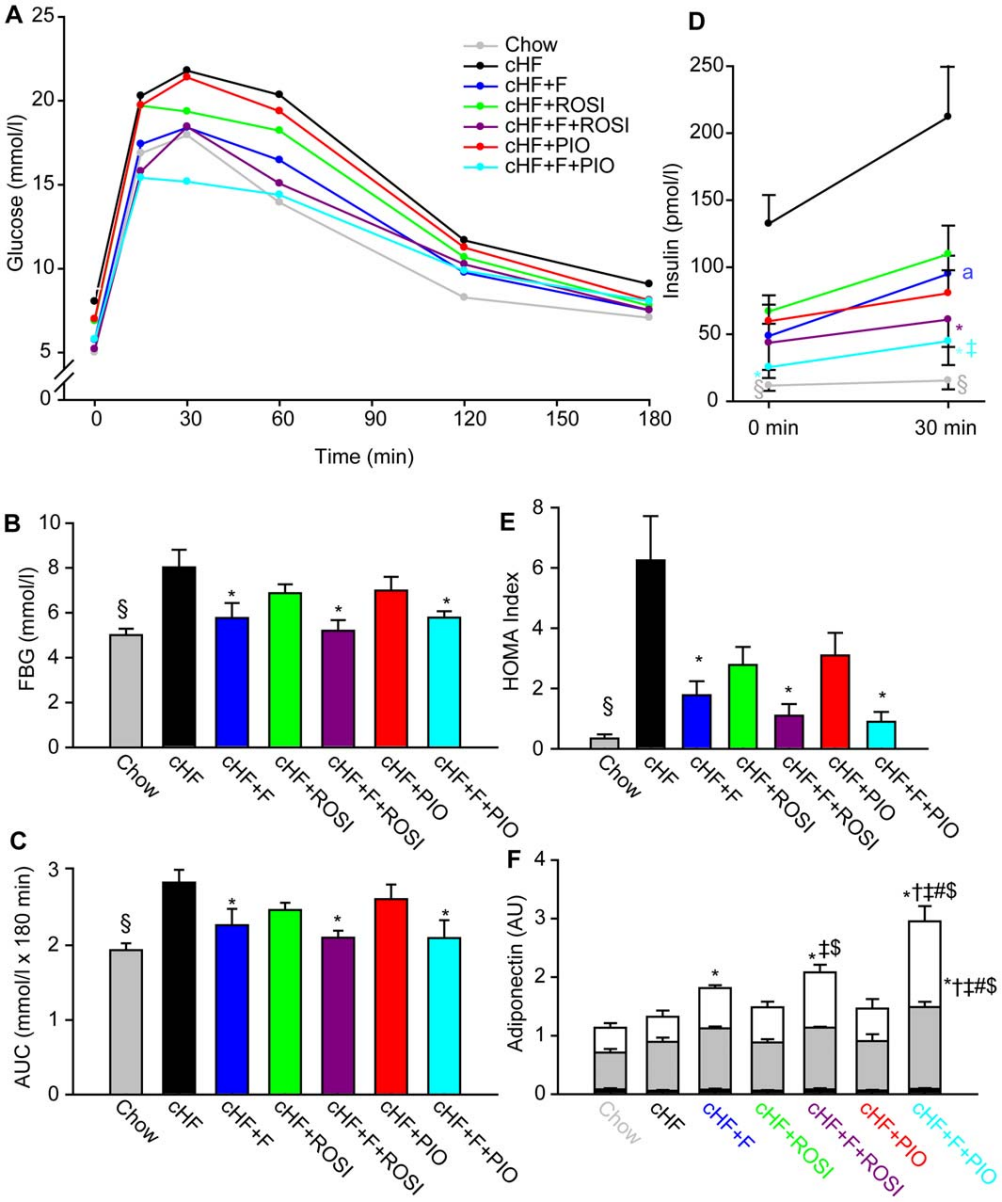
Preservation of glucose homeostasis during high-fat feeding by differential dietary treatment. At 3 month of age, subgroups of mice were placed on cHF diet, or treated various cHF-based diets. During week 7 of the treatment, glucose tolerance test was performed (**A–C**). **A.** Plasma glucose levels following i.p. glucose injection (time 0) to mice fed by Chow or cHF diet, or treated by cHF+F, cHF+ROSI, cHF+F+ROSI, cHF+PIO, and cHF+F+PIO diet. **B.** Fasting blood glucose levels at the baseline (time 0; see **A**). **C.** Total area under the glycaemic curve values as above (**A**). **D.** Insulin levels in plasma (at time 0 and at 30 min); lines and symbols as above (**A**). **E** HOMA index calculated from glucose and insulin plasma levels at time 0. **F.** Adiponectin levels in plasma at time 0; bar height, total immunoreactive adiponectin; black section, low molecular weight adiponectin; gray section, medium molecular weight adiponectin; white section, high molecular weight adiponectin; corresponding SE values are indicated. **B–F.** Data are means±SE (*n* = 7–8). ^a^ significantly different between 0 min and 30 min. *Significantly different from cHF; ^†^significantly different from cHF+F; ‡significantly different from cHF+ROSI; ^#^significantly different from cHF+F+ROSI;^ $^significantly different from cHF+PIO (ANOVA). ^§^Significantly different from cHF (t-test). AUC, area under the curve; AU, arbitrary units; FBG, fasting blood glucose.

During week 7 (see Fig. 1), intraperitoneal glucose tolerance test was performed (Fig. 4A,B,C). In accordance with the measurements of blood glucose in the FASTED state (i.e., when food was removed between 8:00 a.m. and 10:00 p.m.) during week 6 (Fig. 3D), baseline fasted glycaemia (time 0; Fig. 4A; food was removed between 5:30 p.m. and 8:30 a.m.) in this case was ∼1.6-fold higher in the cHF mice than in the Chow mice (Fig. 4A,B). This increase was fully prevented by the cHF+F treatment, as well as by the combination treatments (cHF+F+ROSI and cHF+F+PIO), while cHF+ROSI and cHF+PIO treatments tended to had smaller effects. cHF-feeding impaired glucose tolerance (Fig. 4A,C), while the treatments provided a similar kind of protection as in the case of their effect on fasted glycaemia (see above). Plasma insulin was also measured at the baseline and at 30 min after glucose injection (Fig. 4D), when the maximum increase in glycaemia was expected (Fig. 4A). In the cHF mice, at both time points, insulin levels were much higher as compared to the Chow mice, and they also increased in response to glucose. The effect of cHF diet was largely prevented by both combination treatments, while single-type treatments showed smaller effects. Pioglitazone lowered insulin levels more effectively as compared to rosiglitazone, independent of the type of treatment (except for cHF+ROSI vs cHF+PIO at the time 0; Fig. 4D). Changes in the HOMA index supported the superior efficacy of the combination treatments in the preservation of glucose homeostasis (Fig. 4E). Thus, as compared with the preservation of various parameters of glucose homeostasis by the single TZD-treatments (either cHF+ROSI or cHF+PIO), the superior efficacy of the combination treatments was apparent. However, the difference was only significant in case of plasma insulin levels in the cHF+F+PIO-fed mice at 30 min (Fig. 4D). In neither situation, the effect of the combination treatment was significantly ‘better’ as compared with the effect of the single cHF+F treatment.

The above beneficial effects of various treatments on glucose homeostasis could depend, at least in part, on the induction of adiponectin (see Introduction). Indeed, plasma levels of total adiponectin as well as of its biologically active high molecular weight (HMW) form increased in response to cHF+F treatment (Fig. 4F). Even a stronger induction was observed with both combination treatments, and it was significantly stronger in case of the cHF+F+PIO as compared with cHF+F+ROSI treatment. Single-type treatments using either rosiglitazone or pioglitazone had no effect (Fig. 4F). Thus, a remarkable synergism between *n*-3 LC-PUFA and pioglitazone in the induction of adiponectin was found, resulting in ∼2.3-fold and ∼3.6-fold higher levels of total and HMW adiponectin, respectively, as compared to the cHF mice.

**Figure 5:**
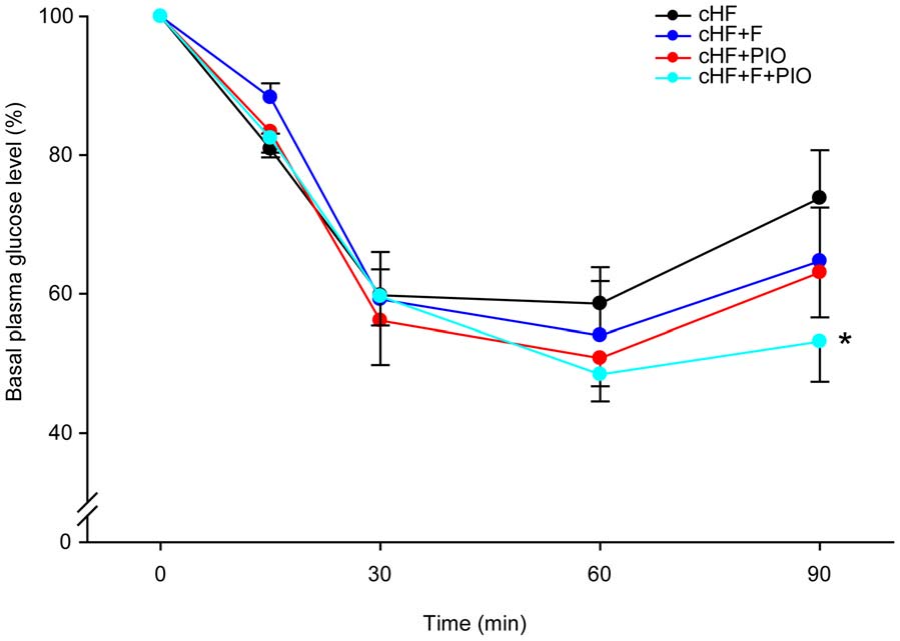
Reversal of insulin resistance in obese mice by differential dietary treatment. Mice were fed cHF diet between 3 and 7 months of age and then fed cHF diet, or treated by cHF+F, cHF+PIO, and cHF+F+PIO diet for 2 weeks (*n* = 8). Plama glucose levels in mice subjected to insulin tolerance test are shown. Body weights of the groups were similar at the beginning of the dietary intervention (average body weight cHF, 42.2±1.9 g; cHF+F, 41.9±2.1 g; cHF+PIO, 41.8±2.4 g; and cHF+F+PIO , 41.9±1.8 g), as well as after 2 weeks of the intervention (not shown). Fasting blood glucose level at beginning of the test were similar in all the groups (cHF, 12.4±0.7 mmol/l; cHF+F, 12.4±0.7 mmol/l; cHF+PIO, 10.6±0.6 mmol/l; and cHF+F+PIO, 9.0±0.4 mmol/l). *significantly different from cHF (ANOVA).

In spite of all the beneficial effects of the combination treatments on glucose homeostasis, two important issues remained unclear, namely (i) how insulin sensitivity was affected by the treatments, and (ii) whether the beneficial effects could be demonstrated under the conditions of established obesity and insulin resistance in dietary obese mice, i.e., under the situation mimicking the status of human T2D patients. Therefore, mice were fed cHF diet between 3 and 7 months of age to induce obesity and to impair glucose homeostasis (see [21]), and then fed either cHF diet, or subjected to cHF+F, or cHF+PIO, or cHF+F+PIO treatment for 2 weeks, until performing an insulin tolerance test (Figs. 1 and 5). During 30 min following insulin injection, a uniform decline of glycemia was observed in all the groups, and at 60 min after the injection, differences between groups started to appear but they were not statistically significant. At 90 min after the injection, when glycemia in all the groups tended to return to pre-injection levels, the cHF+F+PIO mice showed significantly lower blood glucose leves as compared to the cHF mice, and both groups subjected to single treatment (i.e., the cHF+F and cHF+PIO mice) tended to show intermediate glycemia (Fig. 5). Thus, these results documented a reversal of insulin resistance in obese mice in response to the combination treatment using *n*-3 LC-PUFA and piogliotazone, and they are in accordance with the additive/synergistic mechanism of action of the combination treatment (see Discussion).

### Targeted metabolomics

In order to characterise further differential effects of various treatments, plasma concentrations of 163 metabolites (Table S1) providing representative sets of amino acids, sugars, acylcarnitines and phospholipids were measured using flow injection analysis/thermospray mass spectrometry (**FIA-MS**) with Biocrates Absolute*IDQ* ^TM^ targeted metabolomics technology. Plasma samples collected in both FASTED and RE-FED states during week 6 were analysed (see also Fig. 3). Due to a relatively large error of their quantification, 27 metabolites were excluded (Table S1) from all the analyses described below. Partial least squares-discriminant analysis (**PLS-DA**) of all the data separated mice into two distinct groups, reflecting their feeding status, while the effect of the dietary treatment was smaller ( Fig. S1). Following contribution score analysis (Fig. S2), metabolites discriminative between the FASTED and RE-FED states were identified. Most of acylcarnitines increased in response to fasting, while all amino acids levels were elevated in the RE-FED state among all the groups (see also ref. [50]). With a few exceptions, also phosphatidylcholines (namely diacyl-phosphatidylcholines with C30 - C36 side chains, and acyl-alkyl-phosphatidylcholines with C34 - C38 side chains) and lysophosphatidylcholines with C14 - C18 side chains increased in general in response to re-feeding (Fig. S2).

**Figure 6:**
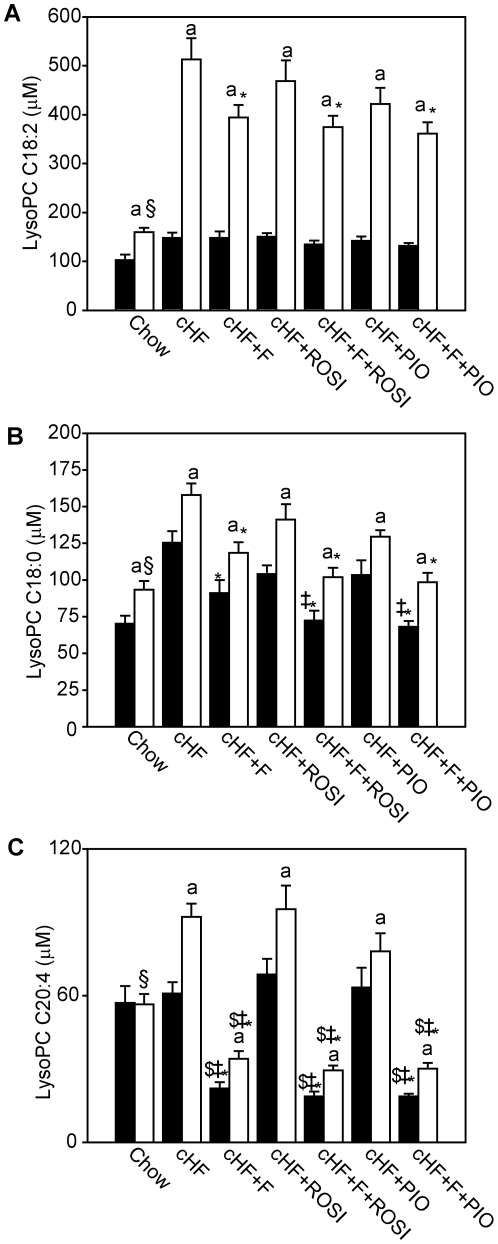
Chemical concentrations of various lysophosphatidylcholines measured in plasma in the FASTED and RE-FED state in all groups of mice. At 3 month of age, subgroups of mice were placed on cHF diet, or treated various cHF-based diets. During week 6 of the treatment, targeted metabolomics analysis was performed. Changes in concentrations of selected analytes are shown (see text, see Fig. 7). **A**. linoleoyl lysophosphatidylcholine (C18:2). **B**. stearoyl lysophosphatidylcholine (C18:0). **C**. arachidonoyl lysophosphatidylcholine (C20:4). Data are means±SE (*n* = 7–8). ^a^significantly different between FASTED (black bars) and RE-FED (white bars) state; *significantly different from cHF; ‡significantly different from cHF+ROSI; ^$^significantly different from cHF+PIO (ANOVA). ^§^Significantly different from cHF (t-test).

Heat map correlation plots revealed stronger separations between treatments in RE-FED (Fig. S3), as compared with the FASTED state (Fig. S4). As expected, metabolomes of the Chow mice and the cHF mice showed vast differences, and also the cHF and the cHF+F mice differed in the concentrations of most lipids determined. However, relatively small differences in metabolome between the cHF+ROSI and the cHF+PIO mice, as well as between the cHF+F+ROSI and the cHF+F+PIO mice were observed (Fig. S3 and Fig. S4). Several metabolites could be unequivocally identified, which showed meaningful changes in their concentrations across all the groups. Namely the contents of C18:2-, C18:0- and C20:4-lysophosphatidylcholine acyls, representing 3 out of 6 most abundant lysophosphatidylcholine acyl species present (Table S1), were higher in the cHF mice than in the Chow mice, especially in the RE-FED state (Fig. 6A,B,C). This elevation was compromised by all the treatments, in accordance with the known association of the above lipids with obesity-induced low-grade systemic inflammation [51], [52], and suggesting amelioration of the inflammatory state by the treatments. The combination treatments tended to exert the most pronounced effects (Fig. 6A,B,C). The content of 20:4-lysophosphatidylcholine increased in response to RE-FED state in all mice fed cHF-based diets, but in the Chow mice. Its levels in the cHF+F, cHF+F+ROSI and cHF+F+PIO mice were very low (Fig. 6C).

**Figure 7:**
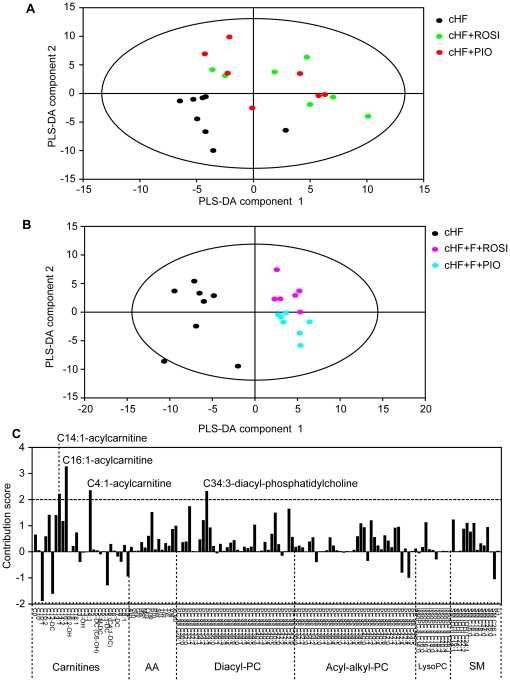
Comparisons of the effects of treatment by TZD-containing diets on plasma metabolome. At 3 month of age, subgroups of mice were placed on cHF diet, or treated various cHF-based diets. During week 6 of the treatment, targeted metabolomics analysis was performed. In total, plasma concentrations of 163 metabolites were determined in both FASTED and RE-FED states during week 6 of the treatment using FIA-MS with the Biocrates Absolute*IDQ* ^TM^ targeted metabolomics technology. After removal of unstable metabolites (see Table S1), 136 metabolites were included in a partial least squares-discriminant analysis (**PLS-DA**), using delta values (**DV**) calculated as a difference in the concentration (***c***) of each metabolite between RE-FED and FASTED state in individual mice (DV  =  RE-FED*_c_* – FASTED*_c_*). 2D-scatter plots of the first (axis X) and the second (axis Y) PLS-DA component are shown for selected groups of mice (*n* = 7–8). **A.** Mice fed cHF diet (black circles), or treated using cHF+ROSI (green circles) or cHF+PIO diet (red circles). **B.** Mice fed cHF diet (black circles), or treated using cHF+F+ROSI (violet circles), or cHF+F+PIO (cyan circles) diet. **C.** Contribution scores for the separation between the cHF+F+ROSI and cHF+F+PIO treatments (see **B**) for each metabolite are shown. Positive value of the score corresponds to a larger DV of the metabolite in the cHF+F+ROSI as compared with the cHF+F+PIO mice. Metabolites with contribution scores >2.0 are labeled (for figure in a zoomable format, see Fig. S5; see Table S1 for the full list of the metabolites and the abbreviations). AA, amino acid; PC, phosphatidylcholine; LysoPC, lysophosphatidylcholine; SM, sphingomyeline and hydroxysphingomyeline.

**Figure 8:**
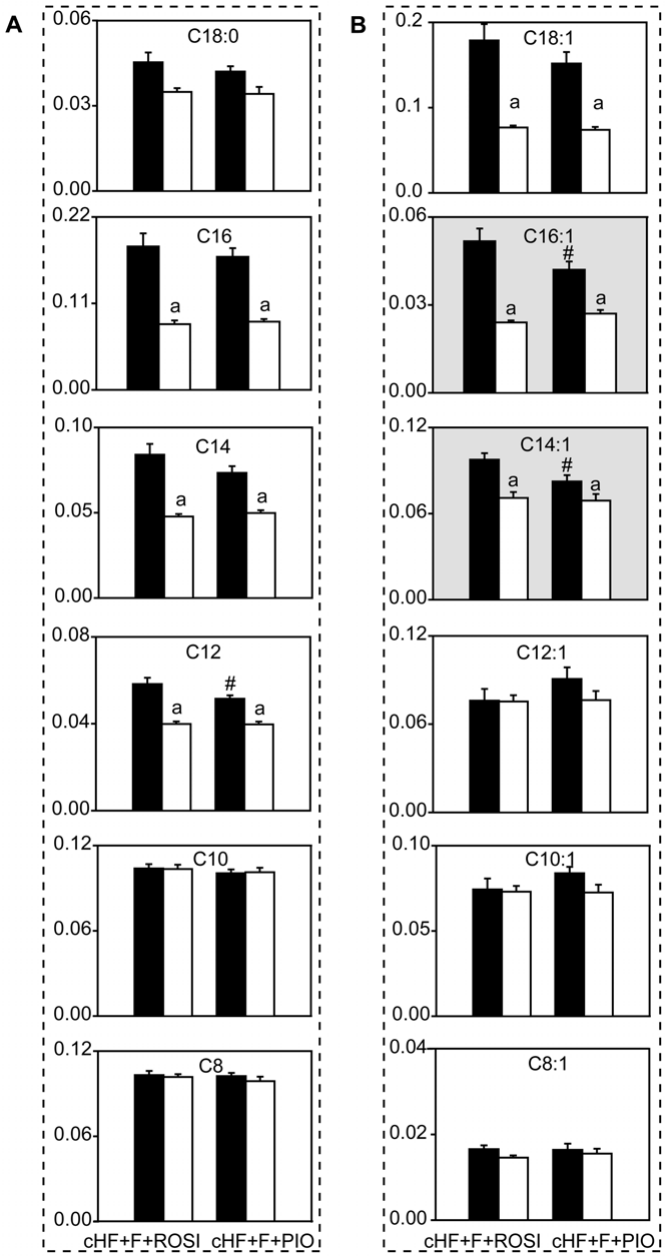
Concentrations of selected even-chain acylcarnitines measured in plasma in the FASTED and RE-FED state in the cHF+F+ROSI and cHF+F+PIO mice. Data are means±SE (*n* = 7–8). A. Saturated acylcarnitines. B. Monounsaturated acylcarnitines. Individual acylcarnitines are denoted by their side chains; see Table S1 for the concentrations all 26 acylcarnitines determined. Axis Y, concentration of each carnitine (µM); gray background, acylcarnitines showing the largest contribution scores (>2) in the separation between the cHF+F+ROSI and cHF+F+PIO mice (see Fig. S5). ^a^significant difference between FASTED (black bars) and RE-FED (white bars) state; ^#^significantly different from cHF+F+ROSI (ANOVA).

The main focus of the analyses was on detailed characterisation of the differential effects of rosiglitazone and pioglitazone. We hypothesised that the discriminating metabolites might be found based on their response to the FASTED/RE-FED transition, as suggested by this type of analysis performed with plasma NEFA, triacylglycerols, and glucose (see Fig. 3A,B,D). First, the difference in each metabolite concentration between RE-FED and FASTED state (**DV**) was analysed using PLS-DA, reflecting the diet, in the cHF, cHF+ROSI and cHF+PIO mice. The first (axis X) as well as the second (axis Y) PLS-DA component showed a weak separation between the cHF mice and the TZD-treated mice, however no separation between the cHF+ROSI and cHF+PIO mice could be observed (Fig. 7A), indicating similar changes in the concentrations of most of the measured metabolites during the FASTED/RE-FED transition, independent of the type of TZD. When the cHF, cHF+F+ROSI and cHF+F+PIO mice were analysed, the first PLS-DA component showed a strong separation between the cHF mice and mice subjected to the combination treatments. Importantly, in contrast to a lack of separation between the single-TZD-treatments, the second PLS-DA component showed a separation between two combination treatments (Fig. 7B). Therefore, these data were further analysed to identify metabolites discriminating between the cHF+F+ROSI and cHF+F+PIO mice. Contribution scores indicated that namely various acylcarnitines contributed to the separation of these two combination treatments (Fig. 7C and Fig. S5). Even side-chain species acylcarnitines in plasma, and especially those with longer chains, reflect activity of mitochondrial β-oxidation [50], [53], [54]. Acylcarnitines with saturated (Fig. 8A) and monounsaturated (Fig. 8B) even side-chains C12-C18 showed lower levels in RE-FED as compared with FASTED state, and the response to FASTED/RE-FED transition of these lipids discriminated between cHF+F+ROSI and cHF+F+PIO treatments, while tetradecenoylcarnitine (C14:1) and palmitoleylcarnitine (C16:1) represented the major discriminating metabolites (Fig. 8B), in fact 2 out of 4 metabolites with the contribution scores >2 (Fig. 7C and Fig. S5). The majority of metabolites including acylcarnitine showed a bigger response to FASTED/RE-FED transition in the case of cHF+F+ROSI as compared to cHF+F+PIO treatment. Levels of acylcarnitines with shorter even side-chains (C<10) were not affected by the feeding status (Fig. 7A,B and Fig. S5).

## Discussion

We have shown that a long-term treatment combining *n*-3 LC-PUFA and either rosiglitazone or pioglitazone markedly improved systemic markers of lipid and glucose homeostasis in mice fed a high-fat diet. However, differences between the effects of rosiglitazone and pioglitazone became apparent under the conditions of the combination treatment, especially with respect to the unique anti-obesity effect of the treatment using rosiglitazone, and relatively strong insulin-sensitizing and hypolipidaemic effects of the pioglitazone-based treatments.

We have confirmed our previous finding [21] that the combination treatment using relatively low doses of rosiglitazone and *n*-3 LC-PUFA exerted surprising additive effects in the prevention of body weight gain, namely due to a reduced accumulation of subcutaneous fat. This is in contrast with the enhanced accumulation of subcutaneous fat in diabetic patients treated with rosiglitazone [9], as well as in mice treated by rosiglitazone using the doses much higher than here (e.g. refs. [14], [15] and our previous study [21]) and eliciting a strong insulin-sensitizing effect [14]. At the dose used in this study, rosiglitazone had no effect on hepatic insulin sensitivity [21]. In a sharp contrast with rosiglitazone, pioglitazone did not affect body weight and adiposity when used in the combination treatment. These results were confirmed by an independent experiment when EPA and DHA were administered as phospholipids rather than triacylglycerols (Rossmeisl et al, unpublished).

The differential anti-obesity effect of the two TZDs in the combination treatments correlated with changes in plasma levels of acylcarnitines with long saturated and monounsaturated even side-chains (C12-C18) in response to re-feeding. Plasma levels of these lipids correlate with the activity of β-oxidation, mainly in skeletal muscle [50], [54], and also in the liver [55], while they are suppressed by insulin, thus reflecting the switch between fatty acid and carbohydrate catabolism [53], [56]. High-fat diet-induced insulin resistance in skeletal muscle is characterised by an impaired switching to carbohydrate oxidation during the FASTED/RE-FED transition, which is mirrored by smaller changes in the tissue levels of long-chain acylcarnitines [50]. Accordingly, the stronger suppression of plasma levels of the long-chain acylcarnitines in response to re-feeding in the cHF+F+ROSI as compared with the cHF+F+PIO mice suggest that the anti-obesity effect of the cHF+F+ROSI treatment reflects the relatively strong stimulation of β-oxidation. The anti-obesity effect was also reflected by the response of NEFA levels in plasma to the FASTED/RE-FED transition. Negligible effects of the treatments on the plasma β-hydroxybutyrate levels, independent of the metabolic status (Table S1), suggest that lipid catabolism in the muscle rather than in the liver was affected differentially by two types of combination treatments. That the combination treatment using *n*-3 LC-PUFA and rosiglitazone specifically induced whole body lipid catabolism was not expected, based on the effects of single-type treatments using rosiglitazone or piogliazone [23], [24]. Thus, the combination treatment could unmask interactions, which caused strong and unexpected biological effects.

The changes in plasma metabolome indicated amelioration of systemic inflammation associated with obesity, namely by the combination treatments, as documented by decreased levels of several lysophosphatidylcholine species, observed also before with both fish oil feeding [51], [52] and metformin therapy in humans [57]. In contrast to linoleoyl lysophosphatidylcholine (C18:2) and stearoyl lysophosphatidylcholine (C18:0), arachidonoyl lysophosphatidylcholine (C20:4) levels were increased by re-feeding only in the dietary obese mice but not in the lean Chow mice, while the suppression of this metabolite levels by *n*-3 LC-PUFA containing diets was very strong, suggesting a replacement of arachidonic acid (**AA**; C20:4*n*-6) in lysophosphatidylcholine by EPA and DHA. Such changes in phospholipid fatty acid composition in obese mice could occur also intracellularly and might have a dramatic impact on eicosanoid formation from PUFA released from membrane phospholipids, while decreasing the formation of pro-inflammatory (AA-derived) and enhancing formation of anti-inflammatory (*n*-3 LC-PUFA-derived) lipid mediators [38], [42], [43], [48].

The additivity in improvement of glucose homeostasis and in preservation of insulin sensitivity was proved previously using cHF+F+ROSI treatment and hyperinsulinaemic-euglycaemic clamps during development of obesity in mice [21]. This was in agreement with the suppression of plasma insulin levels during the glucose tolerance test by both types of combination treatments, which was stronger with pioglitazone as compared to rosiglitazone, also in accordance with the highest induction of adiponectin by the cHF+F+PIO treatment. Moreover, insulin tolerance test perfomed in dietary obese mice in this study suggested that the combined use of *n*-3 LC-PUFA and pioglitazone could provide an additive benefit in reverting insulin resistance. These findings have implications for the treatment of insulin resistance in diabetic patients, concerning the facts that the beneficial effect on insulin sensitivity could be demonstrated under the conditions of established obesity and insulin resistance, and that pioglitazone, i.e. the only TZD approved for clinical use at present, was shown to exert the effect.

In analogy to the situation in humans [10], pioglitazone also exerted stronger triacylglycerol-lowering effects, namely under the RE-FED state. In fact, the cHF+F+PIO treatment resulted not only in the lowest plasma triacylglycerol levels, but also fully prevented the cHF-induced hepatic steatosis, suggesting that (i) the hypolipidaemic effect resulted from the modulation of hepatic lipid metabolism, including the depression of VLDL-triacylglycerols formation, and (ii) the hepatic effects were mediated by AMP-activated protein kinase (AMPK), stimulated by adiponectin. This would be in agreement with the involvement of the adiponectin-AMPK axis in the effects of both TZDs [58] and *n*-3 LC-PUFA [37], with the modulation of hepatic metabolism by *n*-3 LC-PUFA [37] and with the relatively strong activation of hepatic AMPK in response to pioglitazone [22]. The synergistic anti-steatotic effect of the cHF+F+PIO treatment is even more striking in the light of the opposite effects elicited by TZDs, concerning both hepatic steatosis and *Scd-1* expression. We have found previously [20], [21] that SCD-1 activity was stimulated by cHF+ROSI treatment, resulting in elevated formation of palmitoleate and increased hepatic triacylglycerol content. We show here that also pioglitazone, when administered singly, exerts a similar effect on hepatic lipid metabolism as rosiglitazone. However, this effect is less pronounced (see also [22]) and it could be overridden by the combination treatment, documenting potent synergistic interactions between pioglitazone and *n*-3 LC-PUFA, resulting in the pronounced anti-steatotic effect.

In analogy with other combination therapies in the field of metabolic syndrome, namely the combined use of *n*-3 LC-PUFA and simvastatin in subjects treated for hypertriglyceridaemia [3], our experiments on dietary obese mice suggest that also *n*-3 LC-PUFA and TZDs may elicit beneficial additive effects with respect to treatment of impaired glucose tolerance and dyslipidaemia in diabetic patients. Thus in mice, only rosiglitazone but not pioglitazone in the combination with *n*-3 LC-PUFA prevented accretion of body fat, in correlation with the inducibility of fatty acid β-oxidation. However, even in the absence of any effect on body weight, the combination treatment unmasked stronger effect of pioglitazone on glucose homeostasis, triglyceridaemia and hepatic steatosis, depending probably on the induction of adiponectin. It would be interesting to test whether a combination treatment using *n*-3 LC-PUFA and a lower dose of pioglitazone could exert anti-obesity effect or whether a combined use of *n*-3 LC-PUFA and both TZDs could provide a more complex benefit. Importantly, total cholesterol levels in plasma were strongly decreased in response to both TZDs in their combinations with *n*-3 LC-PUFA.

In spite of the concerns related to the adverse side effects of the TZD-based therapy [5], [8], the US Endocrinology Society in its recent statement [61] recommends "to continue the search for additional safe and effective drugs in this class", based on the notion that "adverse events suggests that they might be specific effects related to the individual drug make-up rather than a class effect characteristic of all thiazolidinedione drugs". Accordingly, our results document further that biological effects of various TZD drugs are rather specific, and the differential effects may be unmasked in combination treatments. Our results suggest that *n*-3 LC-PUFA could be used as add-on treatment to pioglitazone, as well as to other prospective drugs from the TZD family (or to structurally diverse PPAR-γ modulators; see [13]), to increase the efficacy of the treatment in diabetic patients and, hence, to decrease the dose requirements of the therapy. The protection against cardiovascular disease mortality of diabetic patients in response to increased *n*-3 LC-PUFA intake [29] represents an important argument in favour of the inclusion of these lipids in the combination treatment of diabetic patients.

## Methods

### Animals and treatments

Male C57BL/6N mice (Charles River Laboratories, Sulzfeld, Germany) were maintained at 22°C on 12-h light-dark cycle (light from 6.00 a.m.) with free access to water and Chow (lipid content ∼3.4% wt/wt; extruded Ssniff R/M-H diet; Ssniff Spezialdieten GmbH, Soest, Germany). Except for the evaluation of insulin sensitivity in dietary obese (see below, see Fig. 5), three-month-old mice (Fig. 1) were randomly assigned (*n* = 8; 2 animals per cage) to cHF diet (lipid content ∼35% wt/wt, mainly corn oil; [21]) or to the following ‘treatments’ by (i) cHF+F, cHF diet supplemented with *n*-3 LC-PUFA concentrate (46% DHA, 14% EPA, wt/wt, as triacylglycerols; product EPAX 1050 TG; EPAX a.s., Lysaker, Norway), which replaced 15% wt/wt of dietary lipids; (ii) cHF+ROSI, cHF diet supplemented with 10 mg rosiglitazone/kg diet (Avandia; GlaxoSmithKline, USA); (iii) cHF+F+ROSI, cHF diet supplemented with both *n*-3 LC-PUFA concentrate and rosiglitazone; (iv) cHF+PIO, cHF diet supplemented with 50 mg pioglitazone/kg diet (Actos; Takeda, Japan); and (v) cHF+F+PIO, cHF diet supplemented with both *n*-3 LC-PUFA concentrate and pioglitazone. During the treatment lasting for 8 weeks (week 1 – week 8; see Fig. 1), fresh ration of food was distributed daily and food consumption and body weights were recorded once a week. Eventually, to analyse all the animals under identical nutritional conditions, mice were fasted during the day (between 8.00 a.m. and 6.00 p.m.), and then allowed free access to Chow during the night and in the morning until the time of killing the animals under pentobarbital anaesthesia (between 9.00 and 11.00 am). Liver and gastrocnemius muscle were dissected and EDTA-plasma was isolated and stored for further analyses. To characterize the effect of the treatment on insulin sensitivity in obese mice, a separate experiment was performed, in which all the animals were fed cHF diet between 3 and 7 months of age, and then singly caged animals were randomly assigned (*n* = 8) to cHF diet, or they were treated by cHF+F, or cHF+PIO, or cHF+F+PIO diet for 2 weeks, i.e. the time when insulin tolerance test was performed (see below).

The animal experiments were specifically approved by the Animal Care and Use Committee of the Institute of Physiology Academy of Sciences of the Czech Republic v.v.i. (Approval Number: 172/2009) and conducted under the guidelines.

### Plasma variables

When indicated, EDTA-plasma was collected using tail bleeding in both FASTED and RE-FED state, as before [59]. Thus, before the bleeding, one half of mice within each experimental group was either (i) fasted for 14 hours (food was removed between 8:00 a.m. and 10:00 p.m., while mice were kept in a clean new cage), or (ii) fasted for 10 hours (between 8:00 a.m. and 6:00 p.m.) and allowed free access to food for the following 3 hours. In each mouse, NEFA, triacylglycerols, leptin and total cholesterol in EDTA-plasma, and glycaemia were assessed as before [21], and plasma levels of β-hydroxybutyrate levels were determined using Autokit 3-HB (Wako Chemicals, Germany, Neuss) with calibration to Ketone Body Calibrator (Wako Chemicals, Germany, Neuss), in both FASTED and RE-FED state, while altering the above protocols during the two subsequent days. Multimeric forms of adiponectin in FASTED plasma were determined using Western blotting [59].

In addition, in 0.01 ml plasma-aliquots collected in both FASTED and RE-FED state, concentrations of 163 metabolites were determined using a metabolomics kit (Absolute*IDQ* ^TM^ kit p150, Biocrates Life Sciences AG, Innsbruck, Austria) based on FIA-MS as before [60]. Concentrations of all analysed metabolites are reported in µM. When indicated, response of each metabolite to the FASTED/RE-FED transition in individual mice was calculated as a difference (**DV**) in the metabolite concentration (***c***) under the two feeding states (DV  =  RE-FED*_c_* – FASTED*_c_*). For the general information on biological roles of the metabolites, see [60]. In short, 14 amino acids, sum of hexoses, free carnitine, 26 acylcarnitines, 14 hydroxy- and dicarboxy-acylcarnitines, 10 sphyngomyelins, 5 hydroxysphyngomyelins, 38 diacyl-phosphatidylcholines, 39 acyl-alkyl-phospatidylcholines, and 15 lysophosphatidylcholines were identified. For the full list of the measured metabolites and the abbreviations to denote them, see Table S1. To ensure the data quality, 27 unstable metabolites were removed, based on the low (≤0.25) coefficient of variance of the same 10 reference samples or large proportion (>10%) of measured samples below the limit of detection (LOD; see Table S1).

### Glucose homeostasis

Intraperitoneal glucose tolerance test was performed in overnight fasted mice (food was removed between 5:30 p.m. and 8:30 a.m., i.e the time of the start of the test by the injection of D-glucose (1 g/kg body weight); ref. [59]), in which glycaemia was assessed using tail bleeds just before the injection (fasting blood glucose at the baseline), and during 180 min after the injection using glucometers (LifeScan, USA). Insulin levels were also determined at the baseline and 30 min after the glucose injection. HOMA index was calculated by the following formula: FASTED plasma insulin (mU/l) x FASTED plasma glucose (mmol/l) / 22.5 [38]. Insulin tolerance test was performed in mice starved for 4 hours (food was removed between 7 a.m. and 11 a.m.). At 0, 15, 30 and 90 min following i.p. injection of insulin (0.75 U/kg; Actrapid, Novo Nordisk, Denmark), glucose levels in tail blood were monitored as above.

### Tissue lipid content and gene expression

Liver and muscle triacylglycerol content was estimated in ethanol KOH solubilisates, and the levels of SCD-1 gene transcript in total liver RNA was evaluated using quantitative RT-PCR [21].

### Statistical analysis

All values are presented as means±SE. Comparisons were judged to be significant at *p*≤0.05 (see Text S1). PLS-DA was performed using Umetrics SIMCA-P+12 statistical software (Umetrics AB, Umea, Sweden; see Text S1).

## Supporting Information

Figure S1 **Comparisons of the effects of feeding status and treatments on plasma metabolome and identification of discriminative metabolites.** In total, plasma concentrations of 163 metabolites were determined in both FASTED (triangle symbols) and RE-FED (circle symbols) states during week 6 of the treatment using FIA-MS with the Biocrates Absolute*IDQ*™ targeted metabolomics technology. After removal of unstable metabolites (see Table S1), 136 metabolites were included in a partial least squares-discriminant analysis (**PLS-DA)**. 2D-scatter plots of the first (axis X) and the second (axis Y) PLS-DA component are shown for all dietary treatments. First PLS-DA component (axis X) separated mice into two distinct groups, reflecting the feeding status and indicating that FASTED and RE-FED states differed substantially in global metabolic profile. The second PLS-DA component (axis Y) showed only a weak separation, with a stronger difference between cHF and chow diet. Different colours were used to indicate the diet and the treatment as in Fig. 1 and Fig. 2.
(TIF)

Figure S2 **The most discriminative metabolites identified using contribution score analysis.** Contribution scores for the separation between the FASTED and RE-FED state using PLS-DA in Fig. S1, independent on the dietary treatment, for each metabolite are shown. For the full list of 163 measured metabolites, 136 metabolites included in the analysis and the abbreviations to denote them, see Table S1. A positive contribution score value indicates higher level of the metabolite in FASTED as compared to RE-FED state (see also Table S1).
(TIF)

Figure S3 **Hierarchial clustering of metabolites in plasma with respect to dietary treatments.** In total, plasma concentrations of 163 metabolites were determined in RE-FED state during the week 6 of the treatment using FIA-MS with the Biocrates Absolute*IDQ*™ targeted metabolomics technology. After removal of unstable metabolites (see ESM Table 1), 136 metabolites were included in the analysis. The ratio of the concentration of each metabolite in each dietary group to a common reference pool is represented by the colour of each cell in the heatmap (green and red, indicates increased and decreased concentration, respectively; see also the Colour Key in the figure). Vertical dendrogram, clustering of metabolites. Each square in the heatmap represents x-fold change relative to mean concentration of each metabolite in all mice (*n* = 7-8) in a color-coded way. Data analysis was performed under the R statistical environment (http://www.r-project.org/). For the full list of 163 measured metabolites, 136 metabolites included in the analysis and the abbreviations to denote them, see Table S1.
(TIF)

Figure S4 **Hierarchical clustering of metabolites in plasma with respect to dietary treatments.** As in Fig. S3, but for FASTED state.
(TIF)

Figure S5 **Contribution of individual metabolites (136 metabolites in total) to the global difference in plasma metabolome between the cHF+F+ROSI and cHF+F+PIO treatments.** In addition to the combination treatment groups, also the cHF mice were included in the partial least squares-discriminant analysis (**PLS-DA**), which was performed using delta values (**DV**) calculated as a difference in the concentration (***c***) of each metabolite between RE-FED and FASTED state in individual mice; see Fig. 7B of the main text. Contribution scores for the separation between the cHF+F+ROSI and cHF+F+PIO treatments for each metabolite are shown (see also Fig. 7C). Positive value of the score corresponds to a larger DV of the metabolite in the cHF+F+ROSI as compared with the cHF+F+PIO mice. Chemical concentrations of acylcarnitines measured in plasma in the FASTED and RE-FED states are shown in Fig. 8 of the main text. For the full list of measured metabolites and the abbreviations to denote them, see Table S1.
(TIF)

Table S1 **List of all 163 metabolites measured in plasma - targeted metabolomics** ***See separate files for the tabulated data (both as a pdf file and an Excel sheet).*** In short, 14 amino acids, sum of hexoses (H1), free carnitine (C0), 26 acylcarnitines (C2, C3,…C18:2), 14 hydroxy- and dicarboxy-acylcarnitines (C-3OH, C4-OH, C5-DC,…C18:1-OH), 10 sphyngomyelins (SM C16:0, SM C16:1,… SM C26:1), 5 hydroxysphyngomyelins [SM(OH)C14:1… SM(OH)C24:1], 38 diacyl-phosphatidylcholines (PC aa C24:0 …PC aa C42:6), 39 acyl-alkyl-phospatidylcholines (PC ae C30:0… PC ae C44:6), and 15 lyso-phosphatidylcholines (lysoPC a C6:0….lysoPC a C28:1) were identified. Plasma concentrations of all analysed metabolites are reported in µM. Gray fonts, 27 unstable metabolites removed from all data analyses, based on the low (≤0.25) coefficient of variance of the same 10 reference samples or large proportion (>10%) of measured samples below LOD. All the metabolites above were determined using flow injection analysis/thermospray mass spectrometry (**FIA-MS**) with Biocrates Absolute*IDQ™* targeted metabolomics technology. In addition, plasma levels of β-hydroxybutyrate levels were determined using Autokit 3-HB (Wako Chemicals, Germany, Neuss) and Ketone Body Calibrator (Wako Chemicals, Germany, Neuss) in both FASTED and RE-FED state. Data are means ± SE (*n* = 7–8).
(XLS)

Text S1 **Statistical analysis.**
(DOC)
