# Supplementary figures and images for: Unmasking Differential Effects of Rosiglitazone and Pioglitazone in the Combination Treatment with n-3 Fatty Acids in Mice Fed a High-Fat Diet
Source: PLoS One. 2011 Nov 3;6(11):e27126. doi: 10.1371/journal.pone.0027126 (PMC3207833; doi:10.1371/journal.pone.0027126)

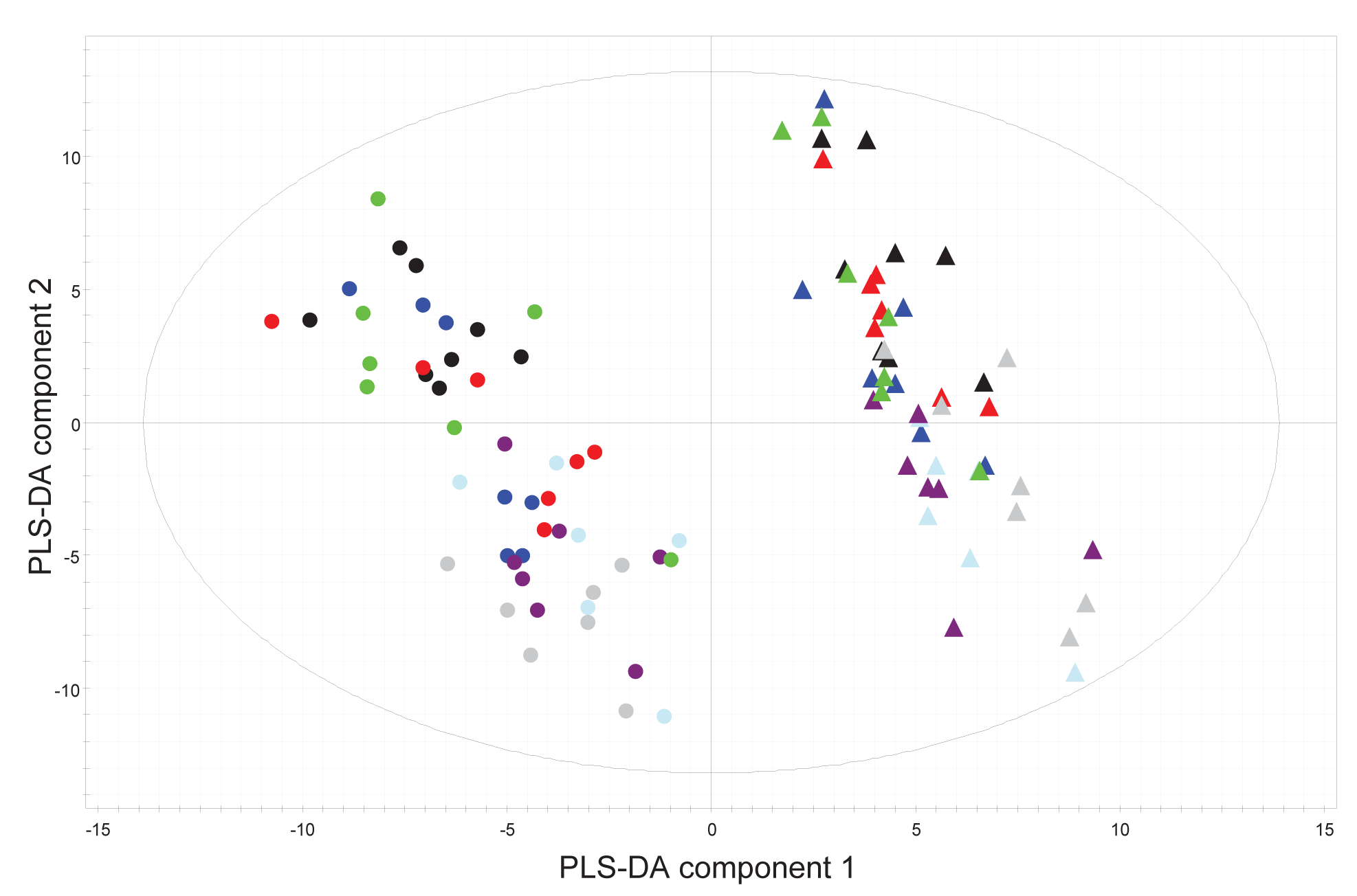

Supplement: Figure S1 — Comparisons of the effects of feeding status and treatments on plasma metabolome and identification of discriminative metabolites. In total, plasma concentrations of 163 metabolites were determined in both FASTED (triangle symbols) and RE-FED (circle symbols) states during week 6 of the treatment using FIA-MS with the Biocrates AbsoluteIDQ™ targeted metabolomics technology. After removal of unstable metabolites (see Table S1), 136 metabolites were included in a partial least squares-discriminant analysis (PLS-DA). 2D-scatter plots of the first (axis X) and the second (axis Y) PLS-DA component are shown for all dietary treatments. First PLS-DA component (axis X) separated mice into two distinct groups, reflecting the feeding status and indicating that FASTED and RE-FED states differed substantially in global metabolic profile. The second PLS-DA component (axis Y) showed only a weak separation, with a stronger difference between cHF and chow diet. Different colours were used to indicate the diet and the treatment as in Fig. 1 and Fig. 2. (TIF) [file pone.0027126.s001.tif]

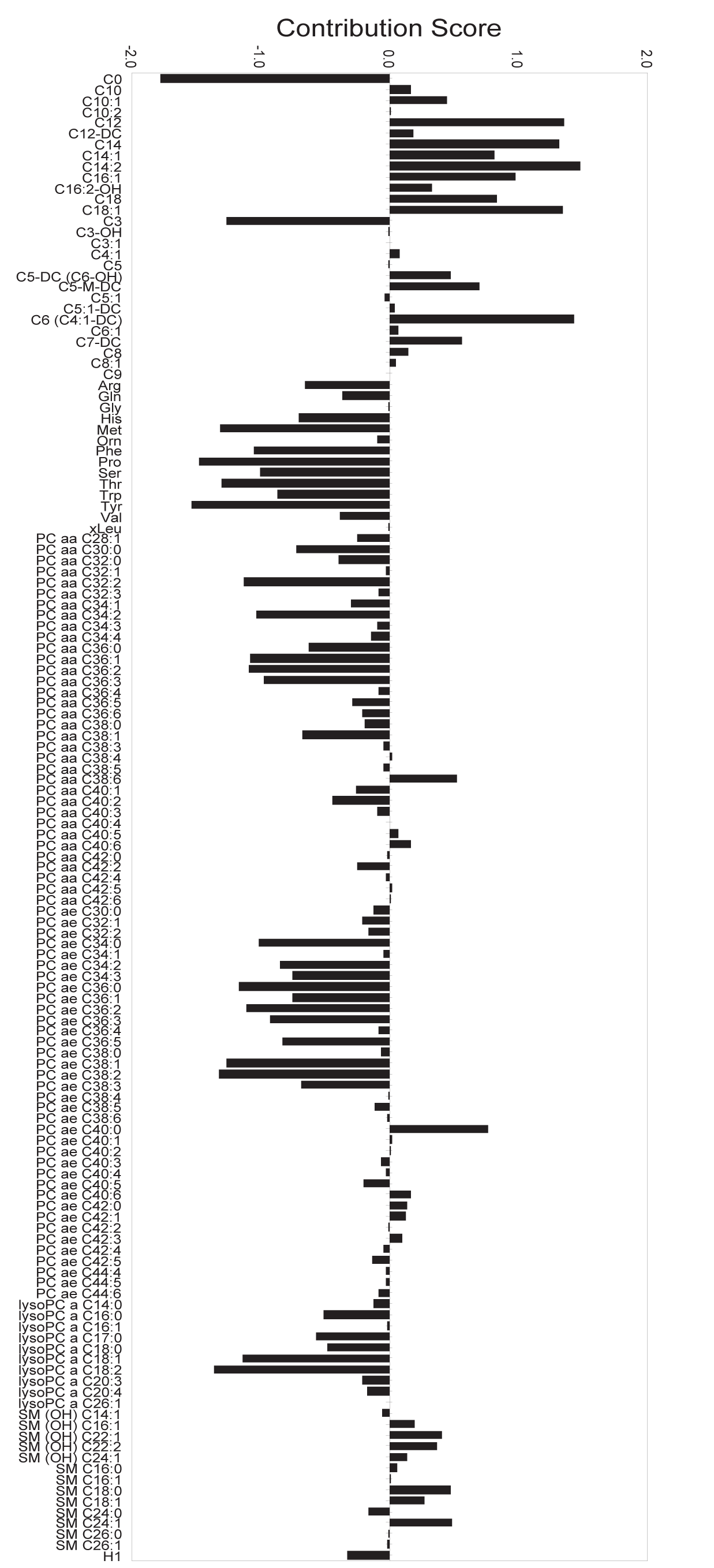

Supplement: Figure S2 — The most discriminative metabolites identified using contribution score analysis. Contribution scores for the separation between the FASTED and RE-FED state using PLS-DA in Fig. S1, independent on the dietary treatment, for each metabolite are shown. For the full list of 163 measured metabolites, 136 metabolites included in the analysis and the abbreviations to denote them, see Table S1. A positive contribution score value indicates higher level of the metabolite in FASTED as compared to RE-FED state (see also Table S1). (TIF) [file pone.0027126.s002.tif]

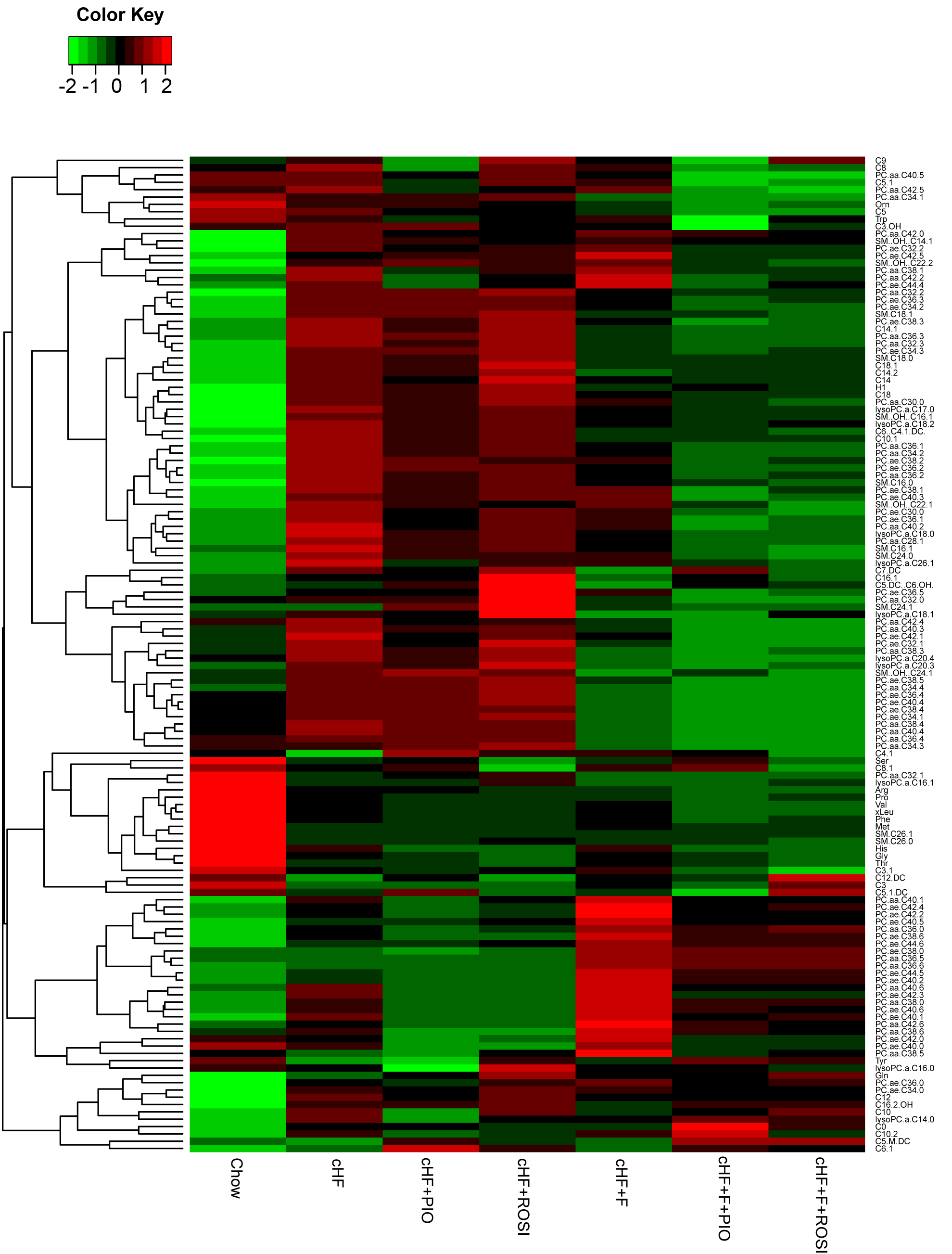

Supplement: Figure S3 — Hierarchial clustering of metabolites in plasma with respect to dietary treatments. In total, plasma concentrations of 163 metabolites were determined in RE-FED state during the week 6 of the treatment using FIA-MS with the Biocrates AbsoluteIDQ™ targeted metabolomics technology. After removal of unstable metabolites (see ESM Table 1), 136 metabolites were included in the analysis. The ratio of the concentration of each metabolite in each dietary group to a common reference pool is represented by the colour of each cell in the heatmap (green and red, indicates increased and decreased concentration, respectively; see also the Colour Key in the figure). Vertical dendrogram, clustering of metabolites. Each square in the heatmap represents x-fold change relative to mean concentration of each metabolite in all mice (n = 7-8) in a color-coded way. Data analysis was performed under the R statistical environment (http://www.r-project.org/). For the full list of 163 measured metabolites, 136 metabolites included in the analysis and the abbreviations to denote them, see Table S1. (TIF) [file pone.0027126.s003.tif]

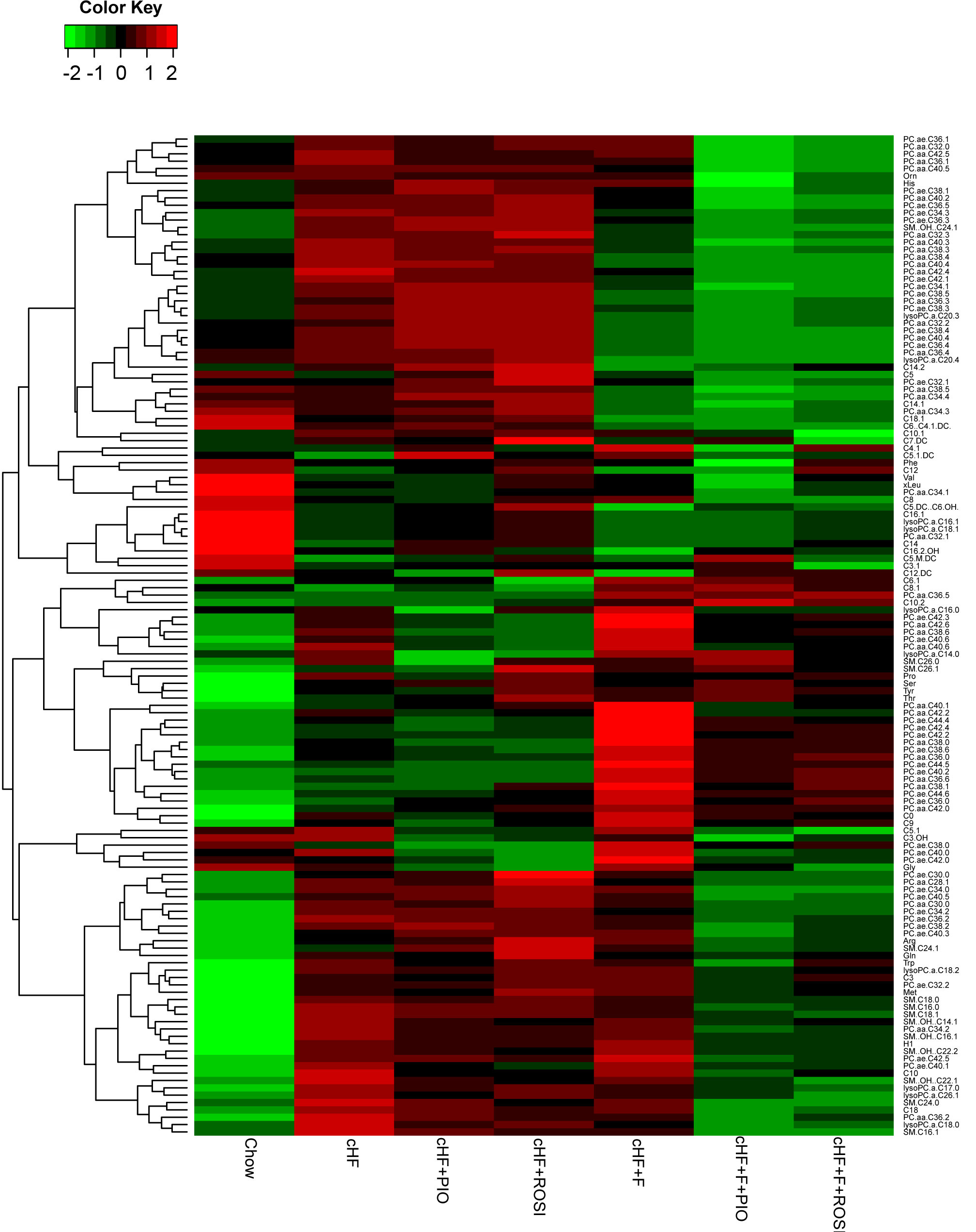

Supplement: Figure S4 — Hierarchical clustering of metabolites in plasma with respect to dietary treatments. As in Fig. S3, but for FASTED state. (TIF) [file pone.0027126.s004.tif]

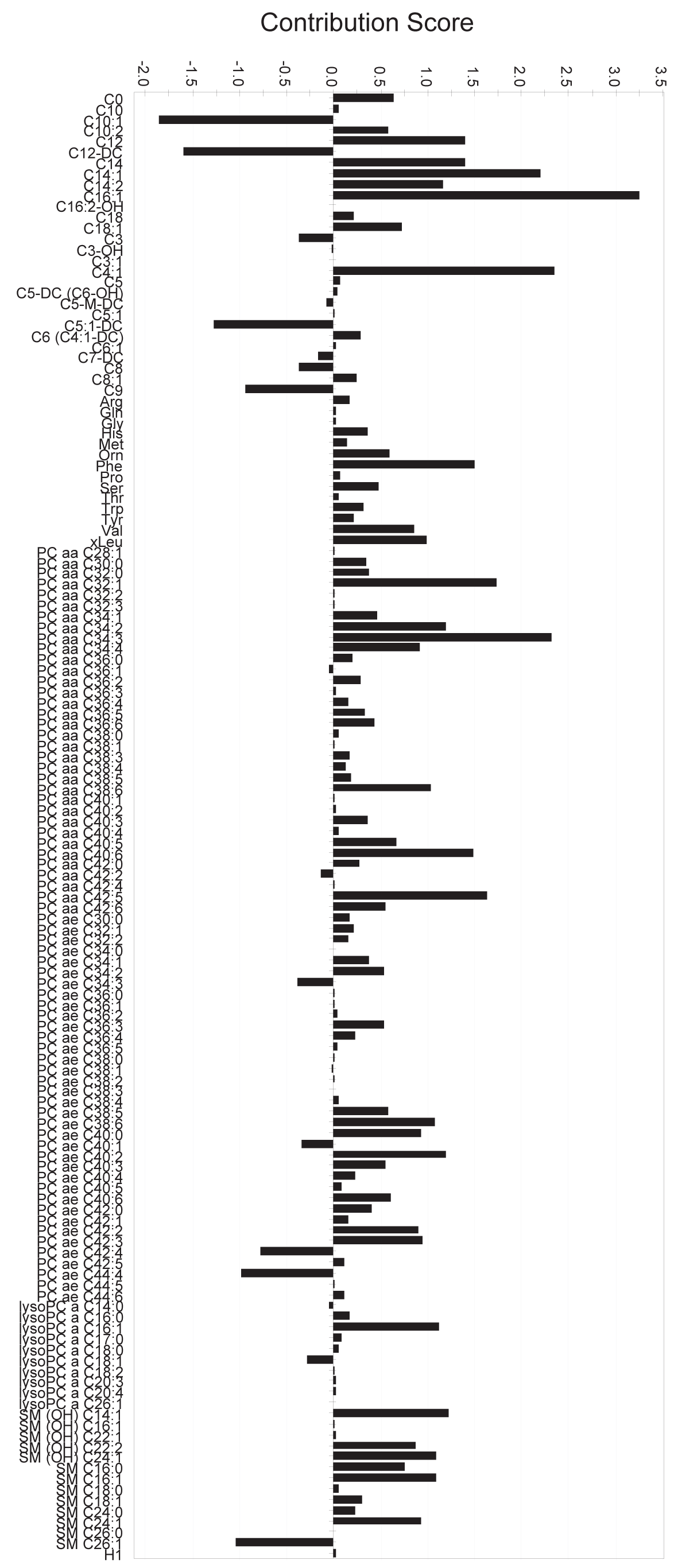

Supplement: Figure S5 — Contribution of individual metabolites (136 metabolites in total) to the global difference in plasma metabolome between the cHF+F+ROSI and cHF+F+PIO treatments. In addition to the combination treatment groups, also the cHF mice were included in the partial least squares-discriminant analysis (PLS-DA), which was performed using delta values (DV) calculated as a difference in the concentration (c) of each metabolite between RE-FED and FASTED state in individual mice; see Fig. 7B of the main text. Contribution scores for the separation between the cHF+F+ROSI and cHF+F+PIO treatments for each metabolite are shown (see also Fig. 7C). Positive value of the score corresponds to a larger DV of the metabolite in the cHF+F+ROSI as compared with the cHF+F+PIO mice. Chemical concentrations of acylcarnitines measured in plasma in the FASTED and RE-FED states are shown in Fig. 8 of the main text. For the full list of measured metabolites and the abbreviations to denote them, see Table S1. (TIF) [file pone.0027126.s005.tif]
