# Supplementary material for: Unmasking Differential Effects of Rosiglitazone and Pioglitazone in the Combination Treatment with n-3 Fatty Acids in Mice Fed a High-Fat Diet
Source: PLoS One. 2011 Nov 3;6(11):e27126. doi: 10.1371/journal.pone.0027126 (PMC3207833; doi:10.1371/journal.pone.0027126)
Supplement: Text S1 — Statistical analysis. (DOC) [file pone.0027126.s007.doc]

**Supporting information**

**Statistical analysis** All values are presented as means±SE. Logarithmic transformation was used to stabilize variance in cells when necessary. Data were analysed by paired *t*-test or ANOVA (one-way or two-way) with Holm-Sidak posthoc tests using SigmaStat 3.5 statistical software. Comparisons were judged to be significant at *p*≤0.05. Repeated measures ANOVA was used to analysis the response of plasma metabolites level to FASTED/RE-FED transition (Fig. 3C,D; Fig. 6). Partial least squares-discriminant analysis (PLS-DA) and SIMCA-P+12 statistical software (Umetrics AB, Umea, Sweden) were used for metabolomic data evaluation. When the PLS-DA score plot showed separated groups, contribution score analysis was performed to determine the variables influencing the separation of the two groups. Results were expressed as a contribution score plot showing one bar per variable, indicating which species deviate most between the groups and in which direction.
